# An Integrated Machine Learning-Based Brain Computer Interface to Classify Diverse Limb Motor Tasks: Explainable Model

**DOI:** 10.3390/s23063171

**Published:** 2023-03-16

**Authors:** Hend A. Hashem, Yousry Abdulazeem, Labib M. Labib, Mostafa A. Elhosseini, Mohamed Shehata

**Affiliations:** 1 Computers and Systems Engineering Department, Faculty of Engineering, Mansoura University, Mansoura 35516, Egypt; 2 Nile Higher Institute of Engineering and Technology, Mansoura University, Mansoura 35516, Egypt; 3 Computer Engineering Department, MISR Higher Institute for Engineering and Technology, Mansoura University, Mansoura 35516, Egypt; 4 College of Computer Science and Engineering, Taibah University, Yanbu 46421, Saudi Arabia; 5 Computer Science and Engineering Department, Speed School of Engineering, University of Louisville, Louisville, KY 40292, USA

**Keywords:** brain–computer interface (BCI), limb motor tasks, whale optimization algorithm (WOA), machine learning classification, BCI competition III dataset IVa, Explainable AI (XAI)

## Abstract

Terminal neurological conditions can affect millions of people worldwide and hinder them from doing their daily tasks and movements normally. Brain computer interface (BCI) is the best hope for many individuals with motor deficiencies. It will help many patients interact with the outside world and handle their daily tasks without assistance. Therefore, machine learning-based BCI systems have emerged as non-invasive techniques for reading out signals from the brain and interpreting them into commands to help those people to perform diverse limb motor tasks. This paper proposes an innovative and improved machine learning-based BCI system that analyzes EEG signals obtained from motor imagery to distinguish among various limb motor tasks based on BCI competition III dataset IVa. The proposed framework pipeline for EEG signal processing performs the following major steps. The first step uses a meta-heuristic optimization technique, called the whale optimization algorithm (WOA), to select the optimal features for discriminating between neural activity patterns. The pipeline then uses machine learning models such as LDA, k-NN, DT, RF, and LR to analyze the chosen features to enhance the precision of EEG signal analysis. The proposed BCI system, which merges the WOA as a feature selection method and the optimized k-NN classification model, demonstrated an overall accuracy of 98.6%, outperforming other machine learning models and previous techniques on the BCI competition III dataset IVa. Additionally, the EEG feature contribution in the ML classification model is reported using Explainable AI (XAI) tools, which provide insights into the individual contributions of the features in the predictions made by the model. By incorporating XAI techniques, the results of this study offer greater transparency and understanding of the relationship between the EEG features and the model’s predictions. The proposed method shows potential levels for better use in controlling diverse limb motor tasks to help people with limb impairments and support them while enhancing their quality of life.

## 1. Introduction

People worldwide suffer from neurological disorders that make it challenging to perform their daily activities. They do not exhibit any changes in their brain activity; however, their limb motor functions are severely compromised. Neuromuscular disorders restrict voluntary muscle movement and speech even while brain ability is unchanged [1]. Many diseases are related to this problem, such as amyotrophic lateral sclerosis (ALS), which affects the nerve cells (i.e., neurons) responsible for controlling voluntary muscle movements. Locked-in syndrome (LIS) is a neurological disorder that rarely happens in which there is a complete paralysis of all voluntary muscles except those that control eye movements [2]. Spinal cord injury (SCI) can leave the affected individuals with paralysis and many other cases [3]. Stroke is a common, potentially fatal neuro-vascular emergency. It is the sixth-highest cause of death globally and one of the main causes of death and disability [4]. Hemiplegia or hemiparesis is the most prevalent and incapacitating condition following a stroke, with up to 30% of survivors having chronic motor impairments [5]. Neurological disorders affect almost one in six people worldwide [6].

In most cases, the disorders above cannot be treated. An alternative would be to provide people with a system enabling them to perform their daily chores independently. Therefore, many researchers started investigating assistive systems, such as brain–computer interfaces (BCIs).

Brain–computer interface (BCI) is a system that can read neurological signals directly from the brain and convert them into an explainable form. A computer or other devices could easily understand this form. Various applications employ it to control things such as wheelchairs, prosthetics, robotic arms, and even word processors [7]. Many people with motor impairments place their hopes in BCI. It will enable many patients to function independently in their daily lives [8]. There are many ways to measure brain signals: non-invasive, partially invasive, and invasive. The most prevalent method, non-invasive, involves attaching electrodes to the outside of the skull to harvest the impulses. This approach provides a good signal quality at a low cost and is simple to apply [7]. One method of this type is electroencephalography (EEG), which records electrical activity along the scalp. EEG measures voltage fluctuations from the ionic current flow within the brain’s neurons [8]. The partially invasive method is the reading of the signals from a device placed on the outside shell of the brain’s grey matter.

On the other hand, the signal strength poses a reduced risk of scarring or damage to the brain’s inside. Electrocorticography (ECOG) is a partially invasive BCI technique in which a thin plastic pad is inserted right above the brain’s cortex [7]. The third technique is invasive, in which the signals should be read from the inside shell of the brain’s grey matter. The latter is a surgical procedure that needs the placement of electrodes within the brain. In theory, this may be the most effective method of gathering data; nevertheless, invasive treatments are not ideal because the human body naturally resists foreign implantation [7].

Motor imaging (MI) is a mental process in which a person imagines completing a movement without doing so or moving the muscles. It is considered the foundation of the majority of BCI systems. It is a dynamic condition in which a certain motor activity’s internal representation is active, but no motor output is obtained [9,10].

Meanwhile, Explainable AI (XAI) is an important area of research in the field of artificial intelligence, as it focuses on developing methods and techniques that make AI models more transparent and understandable. In recent years, XAI has become increasingly relevant for applications in various domains, including medical imaging, natural language processing, and BCIs. In particular, BCIs have the potential to revolutionize the way we interact with technology by allowing us to control devices with our thoughts. However, the development of BCIs requires a deep understanding of the underlying physiological processes, and this requires the use of XAI techniques to provide insights into the behavior of the models used in these applications.

There are five stages for each typical BCI system, including signal acquisition from the brain, signal preprocessing with filters, feature extraction, appropriate feature selection, and classification utilizing suitable classifiers or regression models [11].

The main contributions of this article can be summarized as follows:

- Building a non-invasive brain–computer interface (BCI) based on machine learning to categorize various limb motor tasks.
- The framework uses meta-heuristic optimization for feature selection: whale optimization algorithm (WOA) with multiple hyper-tuned well known machine learning classifiers on a common dataset.
- The interpretable model-agnostic explanations (LIME) technique was used for further explanation of features’ contribution towards the final classification.
- Comparing the latest methodologies to the suggested methods on the same dataset to verify its efficacy shows the proposed strategy’s benefits over others.
- The k-NN machine learning classification model combined with the WOA feature selection approach improves the performance of the BCI system overall.

In this paper, we used the BCI Competition III dataset IVa in several studies using various techniques. We will go into detail about those studies in the following section.

In [12], Attallah, Abougharbia, et al., 2020 used correlation-based feature selection (CFS) and classifier subset evaluation (CSE). They tested the output of this merged technique on various types of classifiers, such as support vector machine (SVM), linear discriminant analysis (LDA), and k-NN. They reported an increase in classification accuracy after performing feature selection. However, despite an increase in accuracy, the system’s accuracy was still not as good as it may have been compared to other methods.

In [13], Molla, Al Shiam, et al., 2020 used the neighborhood component feature selection (NCFS) approach. After applying this method, 30 of the 118 channels were chosen. There are some drawbacks to the process. The duration of MI and the delay in responding to stimuli are subject-dependent, which conflicts with the idea of BCI. The CSP-based features employed in this study are only appropriate for the binary class and cannot be applied to any other classification issues.

In [14], Joadder, Myszewski, et al., 2019 used a method wherein the developed methods were trained and validated using data from all subjects from BCI Competition III dataset IVa. Each time the highest-performing feature was combined with more features, the classification accuracy decreased, indicating that the additional characteristics increased the amount of duplicate information. They concluded that the best feature/classifier mixture for classifying the motor imagery signals is the sample median value combined with the k-NN classifier. Because of this, they achieved high accuracy by using many characteristics.

In [15], Baig, Aslam, et al., 2017 suggested differential evolution (DE)-based EEG feature selection successfully, which offers an excellent feature subset. While all other subjects have classification accuracy above 90%, the subject “av” has the lowest classification accuracy by this method, at 88.9%. However, the suggested approach is slow compared to the common feature selection algorithms, and the wrapper technique’s classifier makes it even slower. SVM and LDA proved to be the best classifiers.

In [16] (Kevric and Subasi 2017), in this study, the maximum classification accuracy was achieved by combining MSPCA de-noising with higher order statistics (HOS) features taken from wavelet packet decomposition (WPD) sub-bands. However, for any of the five subjects from BCI Competition III dataset IVa, according to the authors, the suggested method does not provide the highest categorization precision. However, the single subject, “aa”, for which this suggested method obtains the highest categorization accuracy, was 96%.

In [17] (Dai, Zheng et al., 2018), TKCSP that combines transfer kernel learning and kernel common spatial patterns (KCSP) (TKL) was used in this research. The main advantage of using TKCSP is that it can evaluate various cluster architectures and automatically match them across multiple domains. However, despite using TKCSP in this method, the accuracy was less than many other approaches.

For more information about the previous techniques, Table A1 in Appendix A summarizes these studies that utilized the exact BCI Competition III dataset IVa [18], the methodologies employed in each article, and a remark on each.

The research gap in this study lies in the fact that, despite the dataset utilized being well known and commonly used in other research publications, previous studies have not consistently achieved the highest possible classification accuracy. To address this gap, we developed a brain–computer interface (BCI) classification system that combines a whale optimization algorithm (WOA) for feature selection and multiple classifiers for more accurate classification. Specifically, the developed BCI system was applied to dataset IVa from the BCI Competition III. It should be noted that the limitation of this study is that it is based on a specific dataset, dataset IVa from the BCI Competition III, which may not be representative of other datasets. Furthermore, the proposed BCI system is focused on the specific task of motor-imagery-based classification, which might not generalize to other types of tasks. Future research should investigate the proposed approach on different datasets and different kinds of tasks to validate its generalizability [18], which was shown to be promising. However, to the authors’ knowledge, no other research study has ever produced such classification performance for all patients that can outperform the obtained results by the proposed approach.

## 2. Materials and Methods

### 2.1. Datasets

**Figure 1:**
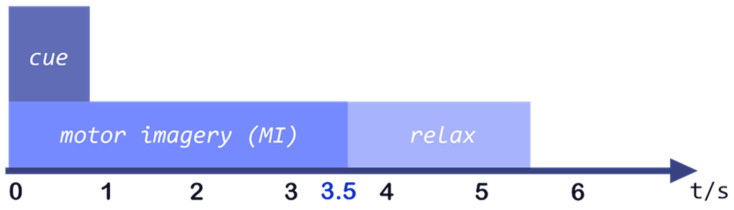
The timing scheme of each trial of data illustrates the data collection process.

The effectiveness of the proposed machine learning-based BCI system will be evaluated by utilizing one publicly accessible data set IVa of BCI Competition III [19], which is based on MI and small training sets. The dataset used in this study is not proprietary, instead, it is a publicly available dataset. How the data were generated is beyond our control, and the dataset is utilized as-is. However, it should be noted that this dataset is widely used as a benchmark for evaluating proposed algorithms. The EEG signals obtained during MI tasks are included in these datasets. The dataset IVa for the BCI competition III encompasses five healthy volunteers (aa, al, av, aw, and ay). The data collection process mainly consists of the following steps: (1) volunteers were settled into a cozy chair, (2) using 118 channels, the EEG data from the five participants were gathered, (3) subjects were instructed to perform MI tasks such as right-hand movement (RH) and foot movement (F) during the EEG recording, (4) each individual was given a specific MI task to complete for 3.5 s, which was communicated by visual cues resulted in a total of 280 signs, and, (5) between each next visual sign, individuals were given brief intervals of about 2 s. The experimental design of each trial is described in Figure 1 [19].

BrainAmp amplifiers and an ECI 128 channel Ag/AgCl electrode cap were used in the recording process. Electrodes were fastened to the scalp utilizing the global 10–20 approach to identify where electrodes should be placed on the scalp [20]. An amount of 118 EEG channels were measured at positions of the extended international 10/20-system. Signals were bandpass filtered between 0.05 and 200 Hz and then digitized at 1000 Hz with 16-bit (0.1 µV) accuracy. In the competition, the data were provided in two versions, and 100 Hz signals were employed. For each individual, the trials were divided unevenly between training and evaluation trials, as demonstrated in Table 1. The reader is directed to refer to [19] for a more comprehensive understanding of the data.

Two classes of epochs from BCI Competition III IVa were gathered from five different courses. EEG signals in each period total 68 channels. The training set receives 80% of the total data gathered from the individuals, while the test set receives 20%. Each subject has a total of 280 epochs, and following the augmentation process, the number of epochs in the training set is multiplied by ten. As a result, each subject’s training and test sets had 10 × 280 × 0.8 and 280 × 0.2, respectively.

### 2.2. The Proposed BCI Approach

**Figure 2:**
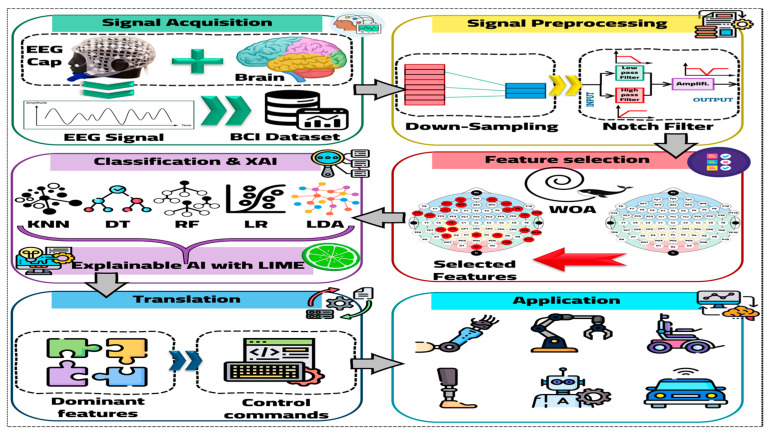
Graphical abstract of the proposed framework.

This paper proposes an innovative and improved machine learning-based BCI system that analyzes EEG signals obtained from MI to distinguish among various limb motor tasks based on the dataset above—starting from signal acquisition, which is the process of recording neural activity. One of the most popular data acquisition techniques utilized in BCI systems is EEG due to its portability, affordability, and simplicity [21]. Signal preprocessing is used to improve noisy signals and eliminate abnormalities, which may include interference from power lines and body movement [22]. As reported by the dataset owners, a notch filter was already implemented in the system. The suggested framework pipeline consists of the following significant steps: (1) using a WOA feature selection technique to find the ideal discriminating features, (2) modeling numerous well known machine learning classifiers, including LDA, k-NN, decision tree (DT), random forest (RF), and logistic regression (LR), and (3) the Explainable AI (XAI) technique was used for further explanation of features contribution towards the final classification. A schematic illustration of the proposed framework is shown in Figure 2.

(1) Signal Acquisition: capturing the appropriate signals is the initial step in using brain signals for information retrieval. There are three classifications for signal acquisition methods: non-invasive, moderately invasive, and invasive [23].

The most common method is non-invasive, since it is thought to be safer than other procedures and because it is so straightforward and does not require as much surgical intervention as other techniques. Magneto-encephalograms (MEG), functional magnetic resonance imaging (fMRI), and electroencephalograms (EEG) are a few of the most common examples of this technique [23].

(2) Signal Preprocessing: it is challenging to accurately decipher brain signals from EEG signal recordings because of various disturbances and artifacts that can interfere with the signal. Due to the recorded signals’ low signal amplitudes, this noise may have an electrical source or be produced by our bodies. Furthermore, numerous aberrations in EEG recordings, including muscle movement or eye blinking, might add additional noise; hence, noise removal is required for EEG [24]. In this phase, The EEG signals from the BCI competition III-IVa dataset are subjected to a notch filter to remove the power-line interference at 60 Hz. Please be aware that the dataset owners have already implemented a notch filter to the autocalibration and recurrent adaptation dataset. Signals between 0.05 and 200 Hz were bandpass filtered, and then they were digitized with 16-bit (0.1 µV) precision at 1000 Hz. In this investigation, we utilized a 100 Hz downsampled version of the data also offered in the competition [18].

The association between EEG indicators and neurologic prognosis following ischemic stroke has been studied in many EEG studies conducted in medical and healthcare settings. For example, in [25], they used machine learning (ML) algorithms on EEG signals to classify stroke patients and healthy people. In [26], they tried to categorize stroke patients’ and healthy adults’ electrical activity by collecting ambulatory EEG data.

(3) Feature Selection is an essential technique for reducing data dimensionality by removing redundancy, determining the features that are directly related to the output, and improving the performance of any suggested system [27,28,29]. Several feature selection strategies were developed to obtain the ideal subset of characteristics. The methods are generally classified into the filter, wrapper, and embedded methods [27]. Without a learning technique, the filter approach determines a subset of features from large datasets. The wrapper approach utilizes a learning algorithm to evaluate the accuracy of a subset of features while categorization is being performed [30]. Recently, much research has studied soft computing techniques’ role in feature selection [31]. Meta-heuristic or nature-inspired approaches are among the most effective and extensively utilized [32]. Numerous applications, including continuous optimization, discrete optimization, and constrained engineering challenges, have used metaheuristic algorithms to predict near-optimal solutions for real-world problems [33]. Particle swarm optimization (PSO), ant colony optimization (ACO), genetic programming (GP), and the whale optimization algorithm (WOA) are a few examples of metaheuristic algorithms [34]. Many metaheuristics, such as grey wolf optimization (GWO), (GA), (ACO), PSO, differential evolution (DE), and dragon algorithm (DA), have been used to handle feature selection difficulties [35].

Significant features from preprocessed EEG data must be collected, processed, and evaluated to design an effective and precise BCI system capable of executing a desired job, such as operating a wheelchair or performing muscle activity. Therefore, feature selection is critical in BCI systems.

The feature selection process has many advantages:

- The feature space is subject to dimensionality reduction to optimize storage capacity and enhance computational efficiency. This approach conserves storage space and improves the quality of the model produced.
- The process involves the elimination of data that is deemed redundant, unnecessary, or noisy. This is performed to prevent the inclusion of such data that might lead to inaccurate or misleading classification performance.
- A direct effect of data analysis tasks is the reduction of time expenditure for the learning algorithm [29].

In this research, the proposed feature selection method is based on the WOA, a meta-heuristic optimization algorithm inspired by nature and developed by Seyedali Mirjalili and Andrew Lewis in 2016 [36].

Algorithm for whale optimization and its variations: WOA offers beneficial characteristics, including fewer controllable parameters (it only has two main internal parameters), simple implementation, and high flexibility [37]. The humpback whales’ use of bubble nets for hunting inspired WOA. A school of fish swimming near the water’s surface is what humpback whales seek to track. They descend to a depth of around 12 m when they detect the prey, where they start to generate distinctive bubbles in the form of a circle or a “9” to surround the target. They then swim toward the prey while following the bubbles [38]. The WOA mathematical representation is discussed in detail as follows:

Encircling prey: The target prey is thought to be the best possible option by humpback whales as they identify and wrap their prey; thus, they try to go closer to the best solution. The equations below [36] describe how updating position behaves.

(1) $$\vec{D}\;=|\vec{C\;}\cdot \vec{X}{}^{*}\left(\mathrm{jt}\right)-\vec{X\;}\;\left(\mathrm{jt}\right)|$$

(2) $$\vec{X\;}\;\left(\mathrm{jt}+1\right)=\vec{X}{}^{*}\left(\mathrm{jt}\right)-\vec{A\;}\cdot \vec{D\;}$$

where jt denotes the current iteration, $\vec{C\;}$ and $\vec{A\;}$ represent the coefficient vectors, the position vector of the best outcome (prey) so far obtained is characterized by is $\vec{X}{}^{*}$, the whale’s position vector is represented by $\vec{X\;}$, and $\vec{D}$ represents the distance between the position vectors of the whale $\vec{X}$ and the prey $\vec{X}{}^{*}$. The following formulas can be used to compute $\vec{A\;}$ and $\vec{C\;}$ [36]:

(3) $$\vec{A}=2\vec{a\;}\cdot \vec{r\;}-\vec{a\;}$$

(4) $$\vec{C\;}=2\cdot \vec{r\;}$$

where $\vec{r\;}$ is a random vector with a range of [0; 1] and $\vec{a\;}$ reduces linearly from 2 to 0 during iterations (in both the exploitation and exploration phases).

**Figure 3:**
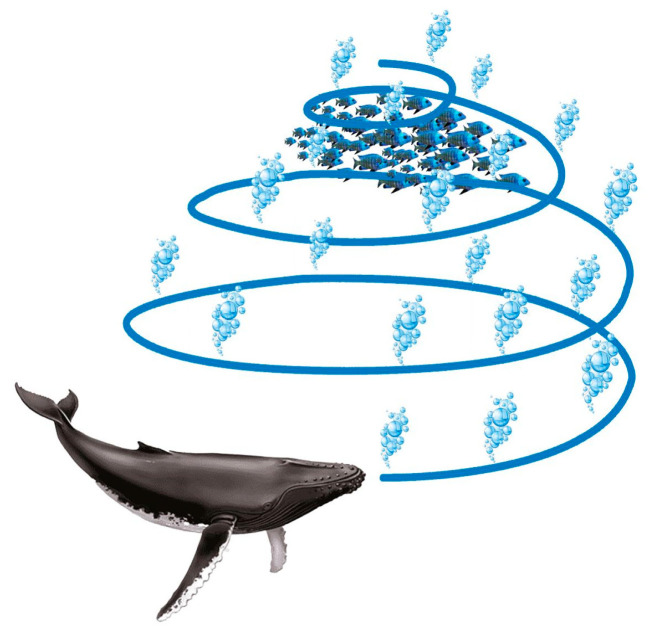
The humpback whales’ bubble-net feeding technique.

The bubble-net attack technique (exploitation phase): Two mathematical models have been created to describe the bubble-net behavior of humpback whales: the shrinking encircling mechanism and the spiral updating position model. Figure 3, which will be detailed in the following section, represents this.

**Figure 4:**
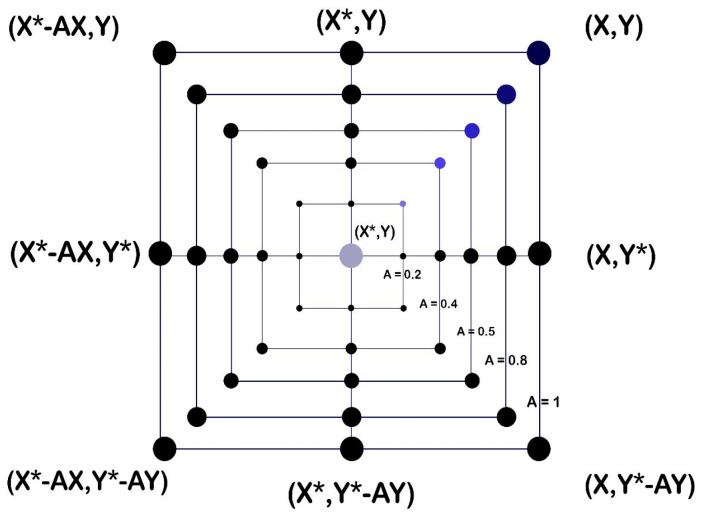
Shrinking encircling mechanism.

Shrinking encircling mechanism: for the achievement of this behavior, the value of $\vec{a\;}$ needs to be decreased in Equation (3) as a result of decreasing $\vec{a\;}$. Additionally, a random value in the range of [−a, a] will be added to reduce the range of $\vec{A\;}$. Over the iteration process, a is reduced from 2 to 0. Using random values for $\vec{A\;}$ in [−1, 1], a search agent’s new position can be defined anywhere between the agent’s starting position and the position of the current best agent. In a two-dimensional space, Figure 4 displays the possible positions from (X, Y) to (X*, Y*) that is achievable by 0 ≤ A ≤ 1.

**Figure 5:**
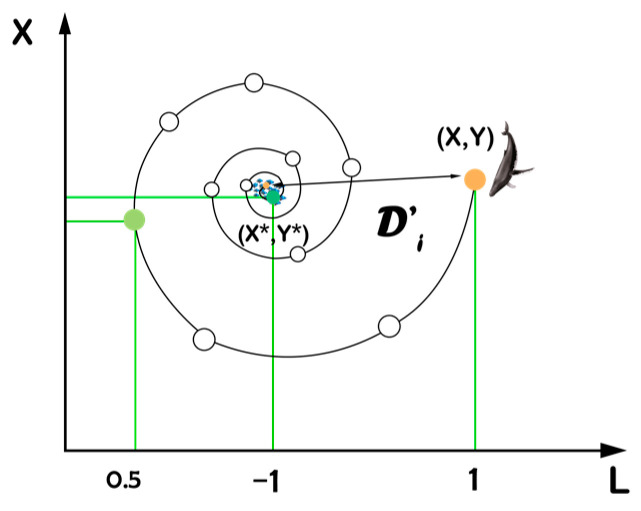
Spiral updating position for bubble-net search.

Spiral updating position: this methodology begins by figuring out how far apart the prey at (X*, Y*) and the whale at (X, Y) are from one another. The following equation establishes a spiral relationship between the position of the whale and its prey to replicate the humpback whales’ helix-shaped movement [38], as seen in Figure 5.

(5) $$\vec{X\;}\;\left(\mathrm{jt}+1\right)=\vec{\overset{`}{D\;}}\;\cdot {\;e}^{\mathrm{bl}}\cdot \;\cos\left(2\pi l\right)+\vec{x^{*\;}}\left(\mathrm{jt}\right)$$

where $\vec{\overset{`}{D\;}}=\left|\vec{X}{}^{*}\left(\mathrm{jt}\right)-\vec{X\;}\;\left(\mathrm{jt}\right)\;\right|$ displays the distance between the ith whale and its prey. The logarithmic spiral’s shape is determined by the constant b.

To update the position of whales throughout optimization and to describe this simultaneous behavior of the whale, we assume that there is a 50% chance of choosing either the spiral model or the shrinking encircling mechanism. The following equation provides the mathematical representation of this assumption [36]:

(6) X → jt+1=x∗ →jt−A →·D → if p<0.5 D `→ · ebl· cos2πl+x∗ →jt if p≥0.5

where p represents a random value in the range [0, 1].

Search for prey (exploration phase): depending on their relative positions, humpback whales randomly search for target. Updating a location depends on $\vec{A\;}$, where A is a random value in the range [−1, 1], and the goal is to make the whale (search agent) move far away from the reference whale. If |A| ≥ 1 is the case and $\vec{X\;}{\;}_{\mathrm{rand}}$ is a random position vector, the search agent’s position is updated as follows [36]:

(7) $$\vec{D}=\left|\vec{C\;}\cdot \;\vec{X\;}{\;}_{\mathrm{rand}}-\vec{X\;}\right|$$

(8) $$\vec{X\;}\;\left(\mathrm{jt}+1\right)=\vec{X\;}{\;}_{\mathrm{rand}}-\vec{A\;}\cdot \vec{D}$$

The pseudocode of the standard WOA’s process phases is shown in Algorithm 1 [36].

**Algorithm 1.** WOA1 Initialize search agents2 Evaluate fitness function.3 jt  →  04 X* = the best possible search agent5 **while** jt < MaxIteration **do**6   **foreach** SearchAgent **do**7    Update A, C, l, p, and a.8    **if**
*p* ≥ 0.5 **then**9      X (jt + 1) = Updating the position of search agent by spiral method in         Equation (5).10   **else**11      **if** |A| < 1 **then**12        X (jt + 1) = Updating position of the current search agent by using            encircling mechanism Equation (2).13      **else if** |A| ≥ 1 **then**14        Random search agent is selected15        X (jt + 1) = Updating position of the current search agent by using prey             searching method in Equation (8).16      **end if**17   **end if**18   **end foreach**19   **If** there is a better solution, update X* = X (jt + 1).20   jt = jt + 121 **end while**22 **return** X*

Transfer function: binary optimization is a subset of feature selection optimization. The feature selection problem can only be solved using binary numbers [0, 1]. As a result, creating a binary variant of the optimization method for usage with the feature selection problem is necessary. According to Mirjalili and Lewis [36], adopting a transfer function (TF) may make it easier to change a continuous optimization method to a binary one. The transfer function converts continuous data into the numbers 0 and 1 based on chance. Equations (9) and (10) are used to implement the s-shape transfer function [33].

(9) $$x_{s2}=\frac{1}{1+e^{-x}}$$

(10) $$X_{\mathrm{bin}}=\left\{\begin{array}{c} 0,\;x_{s2}<N_{\mathrm{random}}\;\\ 1,\;x_{s2}\geq N_{\mathrm{random}} \end{array}\right.$$

where $X_{\mathrm{bin}}$ indicates the feature selection solution issue and $N_{\mathrm{random}}$ is randomly selected as the threshold value.

**Figure 6:**
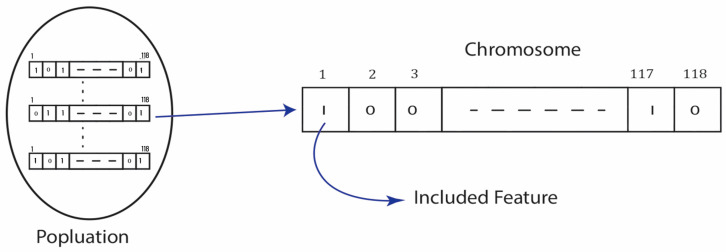
Set of suggested features to be included.

Each feature subset in WOA can be considered a whale’s position. The better the solution, the fewer features there are, and the higher the classification accuracy. Each solution is assessed using the suggested fitness function, which considers two criteria: the accuracy of the solution as determined by the classifier and the total number of features included in the solution [39]. An illustrative example is shown in Figure 6.

**Figure 7:**
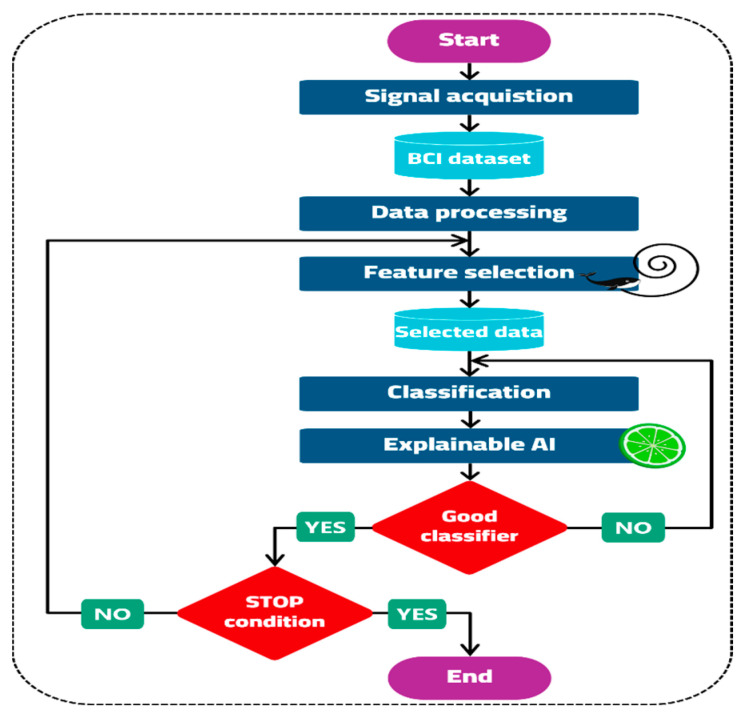
The proposed methodology flow chart.

The sequence of the suggested methodology is illustrated in the flow chart below (Figure 7).

Objective function: the objective function is essential to consider while creating any optimization issue. For instance, wrapper feature selection algorithms minimize the number of features while increasing the accuracy of the learning process. These two opposing goals should be taken into account by the objective function. Equation (11), which uses the selection ratio and the classification error rate as objective functions, was used in this research (minimization) [16].

(11) $${\mathrm{cost}}_{\mathrm{fn}\;}=\;\rho \mathrm{Err}\left(D1\right)+\;\varphi \frac{\left|F\right|}{\left|T\right|}$$

The parameters ρ and φ regulate the feature reduction and classification accuracy, respectively. This result was produced for all datasets in this work using multiple classifiers, and it shows the classifiers error rate of the identified subset. |T| represents the total number of features, and the size of identified feature subset is |F|. In this study, φ is set to 0.99 [30], and φ=1−ρ.

(4) Classification: Following a successful feature selection process, an efficient machine learning-based classification algorithm can analyze and determine the optimal relationship between the input and output attributes to construct a sufficient training model. That training model, if tested, will anticipate the targeted class with an optimal classification performance. Several well known machine learning-based classifiers, such as k-nearest neighbor (k-NN), support vector machine (SVM), random forests (RFs), and artificial neural networks (ANNs), are commonly used for classification purposes.

To evaluate each classifier’s impact and determine the optimum combination, we used a grid search algorithm to hyper-tune several well known machine learning classifiers to obtain the best classification model for the given problem by utilizing classification accuracy as an optimization metric. The classification model demonstrating the highest performance was selected for the proposed BCI system. It is worth mentioning that the WOA was used as a feature selection method for all classifiers. A brief discussion about each classifier is given below.

k-Nearest Neighbor (k-NN) is a supervised learning algorithm based on finding k similar samples in the attribute space [40]. The k-NN algorithm assumes that related things are located nearby. Therefore, the success of the classification depends heavily on the value of k and the distance metric, which must be chosen before using k-NN [41]. Here, we have used a grid search algorithm to obtain the optimal hyper-parameters, where the classification accuracy was used as the optimization metric. The used number of neighbors (k) in this research is 5, and the distance metric was the Euclidean distance.

Two phases form the k-NN classification were finding the closest neighbors comes first and then using those neighbors determines the next class. First, the Euclidean distance metric was used to determine the distance between a target sample and other samples in the feature space, as shown in Equation (12) [42].

(12) $$d\left(p,q\right)=\sqrt{\overset{n}{\underset{i=1}{\sum }}(p_{i}-q_{i}}){\;}^{2}$$

where d(p, q) stands for the distance between the two samples p and q, n is the number of features and $p_{i}$ and $q_{i}$ are the ith feature of the sample.

Random forest (RF): it was developed by Breiman [43], in which it utilizes a classification ensemble learning approach that, during the training phase, employs many decision trees and produces an average prediction of individual trees [44]. In training RF, we set the number of estimators to 100, and the other parameters of the classifier were assigned to the default values criterion“gini”, in_samples_splitint = 2, min_samples_leaf = 1 and max_features “sqrt”.

Linear discriminant analysis (LDA): Figure 4, which will be detailed next, tries to identify the best discriminant qualities by raising the ratio of between-class distance to within-class distance. It has been used with success in various applications [45]. The Bayes theorem is used in the model to calculate the probabilities. This classifier uses the singular value decomposition solver, which is suggested for data with many features. The parameters of the classifier were set to the default values where the solver was ‘svd’ which is recommended for data with a large number of features, shrinkage “none”, prios “none”, n_components “none”, store_covariance “false”, tolfloat = 1.0 × 10^−4^ and covariance_estimator “None”.

Decision Tree (DT) is a tree-structured classifier where each leaf node reflects the result and inside nodes represent the dataset’s features and decision rules [46]. The decision node and leaf node are the two decision tree nodes. While leaf nodes are the results of decisions and do not have any more branches, decision nodes are used to create conclusions and have numerous components. The parameters of the classifier were set to the default values where criterion was set to “gini”, splitter “best”, max_depth “none”, and min_samples_split = 2.

Logistic regression (LR): the class membership probability for one of the two categories in the data set is calculated using the logistic regression model [47]. This method is applied only when classifying binary data. The parameters of the classifier were set to the default values where penalty was ‘l2’, tolfloat = 1.0 × 10^−4^, Cfloat = 1.0, it_interceptbool = True, intercept_scaling = 1, and solver ‘lbfgs’.

(5) Interpretable Model-agnostic Explanations (LIME): LIME is an innovative explanation technique that develops a locally interpretable model around the prediction to explain any classifier prediction in an interpretable and accurate manner [48]. Yet, it is constrained in that it only provides an explanation for a single instance at a time [49]. Here, we used LIME tabular explanations to highlight the contributions of the selected features by WOA to the final output.

## 3. Results

The performance of the proposed machine learning-based BCI system was evaluated on the BCI competition III dataset IVa (please see Table 2) using a variety of measures. However, the accuracy, sensitivity, and specificity are common measurements, which can be calculated as follows [50]:

(13) $$\mathrm{Accuracy}=\frac{\mathrm{Tp}+\mathrm{Tn}}{\mathrm{Tp}+\mathrm{Tn}+\mathrm{Fp}+\mathrm{Fn}}$$

(14) $$\mathrm{Sensitivity}=\frac{\mathrm{Tp}}{\mathrm{Tp}+\mathrm{Fn}}$$

(15) $$\mathrm{Specificity}=\frac{\mathrm{Tn}}{\mathrm{Tn}+\mathrm{Fp}}$$

where true positive (Tp) is a result when the model successfully predicted the positive class. False negative (Fn) results when the model incorrectly predicts the negative class, while true negative (Tn) results when the model incorrectly predicts the negative class. False positive (Fp) results when the model incorrectly predicts the positive class.

For realistic results, and to demonstrate the generalization ability and reproducibility of the proposed system, we tried 20 iterations for each subject. The results were presented at the end in terms of mean ± standard deviation. In addition, results using different hyper-tuned classification models are documented in the following separate Table 3, Table 4, Table 5, Table 6 and Table 7, which correspond to the individual subjects, namely, “aa”, “al”, “av”, “aw”, and “ay”, respectively. The best result was highlighted in bold in the Table 2, Table 3, Table 4, Table 5 and Table 6 below, which represent the outcomes of the classifiers.

**Figure 8:**
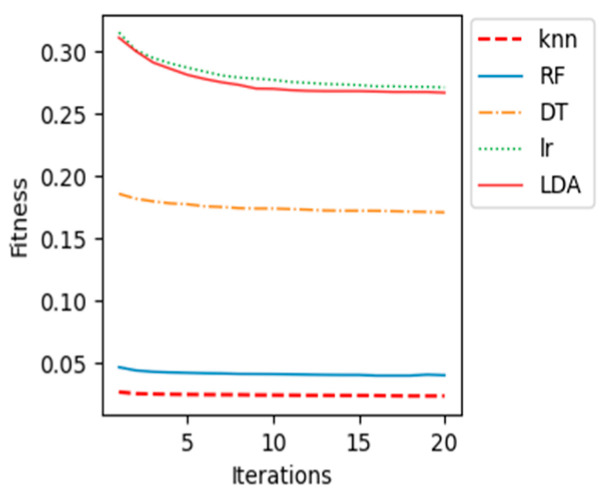
Convergence curve for patient “aa”.

**Figure 9:**
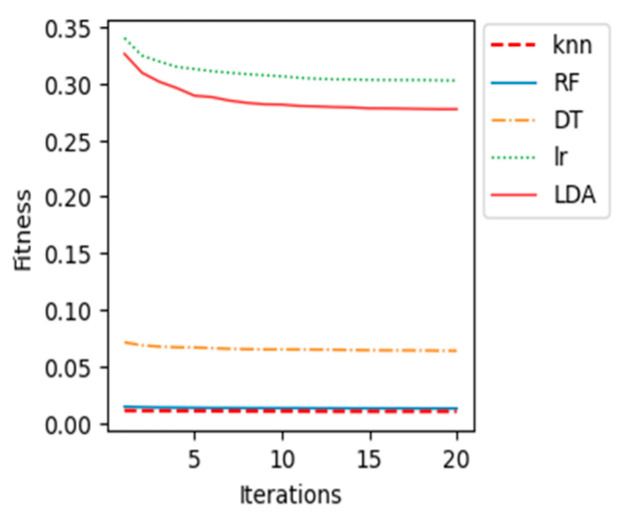
Convergence curve for patient “al”.

**Figure 10:**
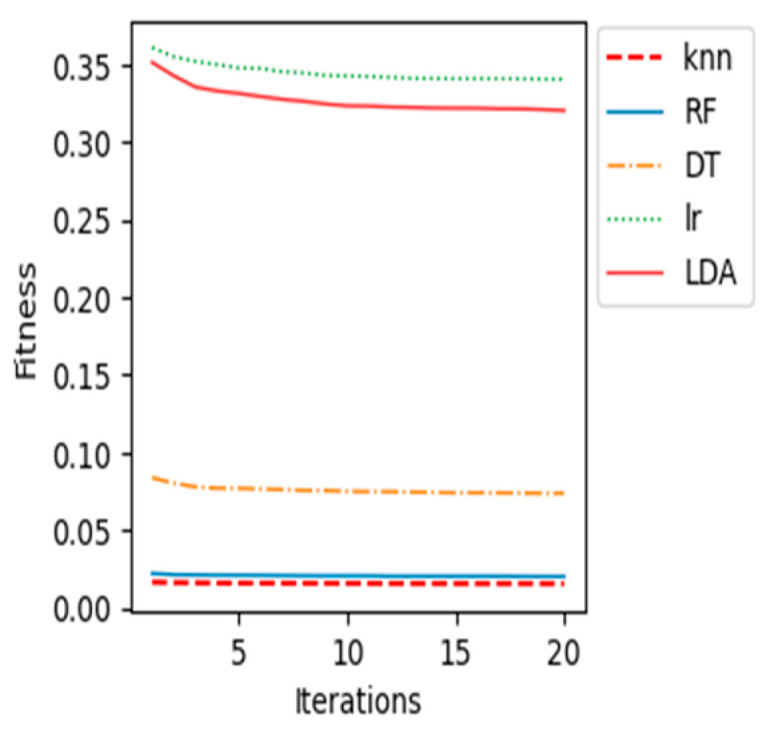
Convergence curve for patient “av”.

**Figure 11:**
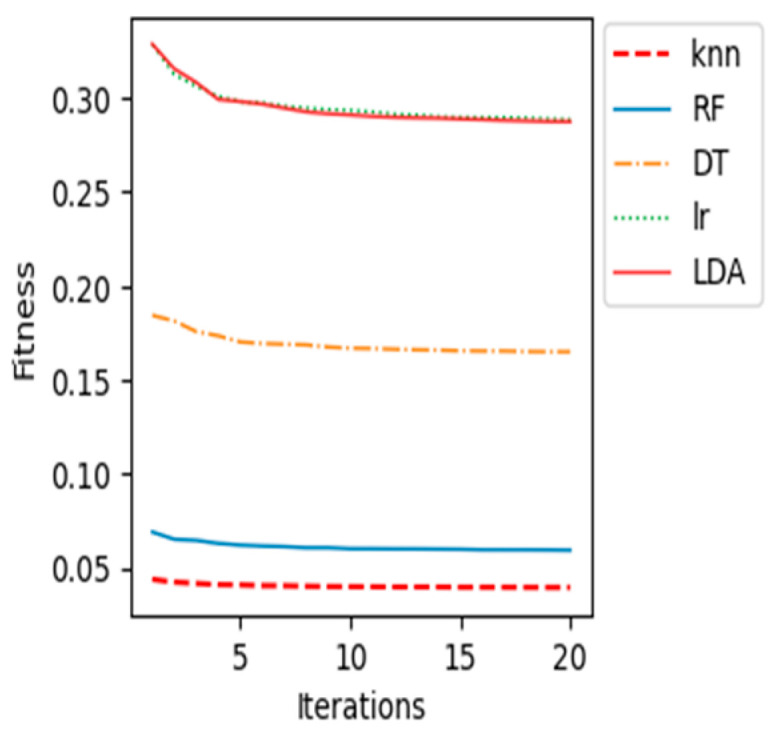
Convergence curve for patient “aw”.

**Figure 12:**
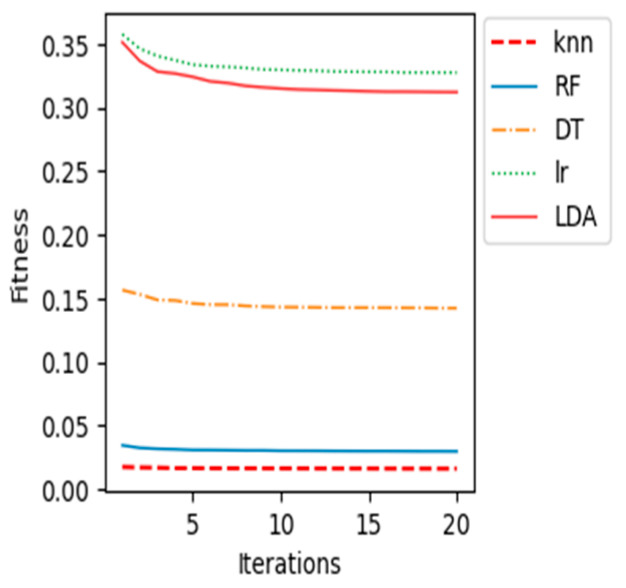
Convergence curve for patient “ay”.

The proposed BCI system, which uses the WOA feature selection algorithm and the k-NN classification model, performed the best out of all the machine learning classification models tested. This is evidenced by its high accuracy, sensitivity, specificity, and AUC. In addition, the following graphs (Figure 8, Figure 9, Figure 10, Figure 11 and Figure 12) show the convergence curve for each patient after applying different machine learning classifiers, which justifies, in particular, the outstanding performance of the k-NN compared with other machine learning classifiers.

The fitness value in the curves is the average fitness value gained from 20 runs for all the used classifiers. In these figures, the proposed k-NN algorithm with WOA is marked with a dashed red line. It is observed that the performance of k-NN was superior for all the patients. The average accuracy of the RF algorithm in patient “al” is quite near to the k-NN results. Although the RF algorithm provided good average accuracy, in all other patients, k-NN results outperformed RF and all different classifiers. As shown in previous curves, the LR algorithm’s average accuracy results are the lowest compared to others.

To demonstrate the benefits of integrating the WOA feature selection technique with the k-NN classification model in the proposed machine learning-based BCI system, Table 7 compares with other studies on the same dataset. The obtained results of the proposed approach outperformed those of the other studies in most subjects (three out of five subjects) and in the overall accuracy of the system, which confirms the outstanding performance of the proposed. The table also included statistical significance *t*-test results to illustrate the significance level between the proposed approach and the other approaches from the literature that used the same dataset.

**Figure 13:**
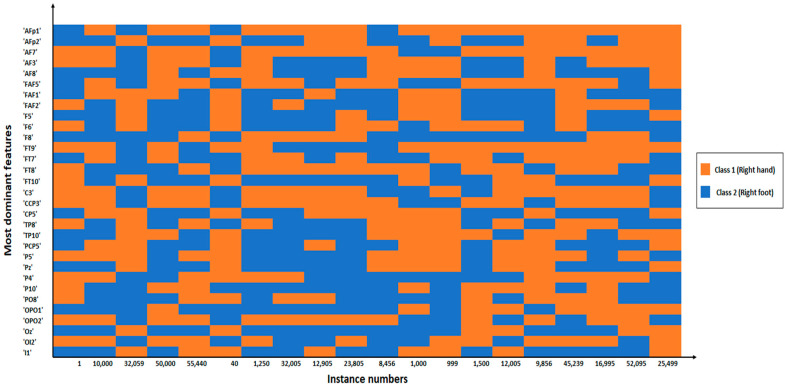
An illustrative heat map shows the contribution for the selected 31 features to each of 20 random instances for being class 1 or class 2.

**Figure 14:**
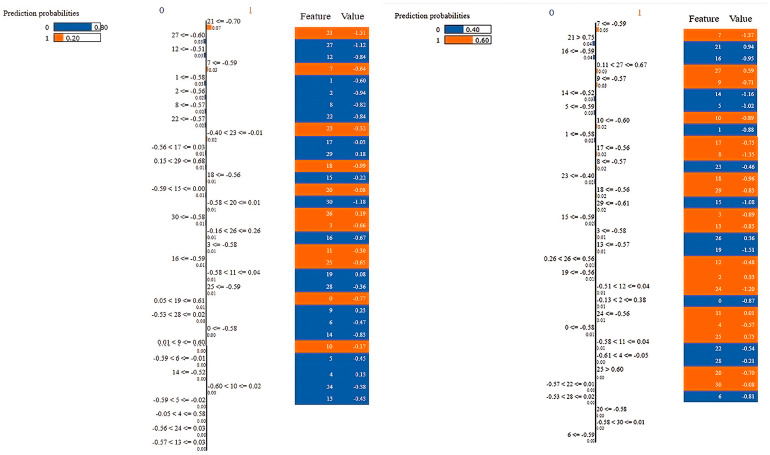
Illustrative examples showing the importance of the contributing selected features with the corresponding values and ranges, while (**left**) an example of an instance from class 1 and (**right**) an example of an instance from class 2.

To generate a list of explanations that show the contribution of each feature to the prediction of a data sample as its output, LIME technique was used, which offers local interpretability and makes it possible to identify the feature changes that have the biggest influence on the prediction. Figure 13 depicts a heat map for a total of 20 instances showing the probability contribution of the selected 31 features by the WOA to randomly selected 10 instances from class 1 (blue) and randomly selected 10 instances from class 2 (orange). Figure 14 is an illustrative example that demonstrates the contribution of the selected features to the probability of being class 1 or class 2. For more details, the reader is referred to Supplementary Materials.

## 4. Discussion

**Figure 15:**
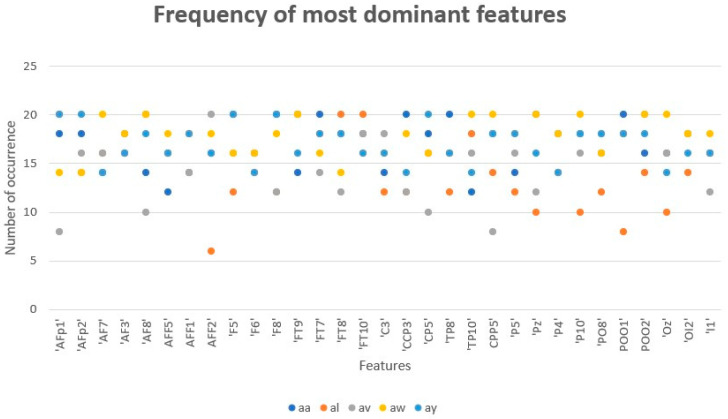
The frequency of the most dominant features across all patients.

**Figure 16:**
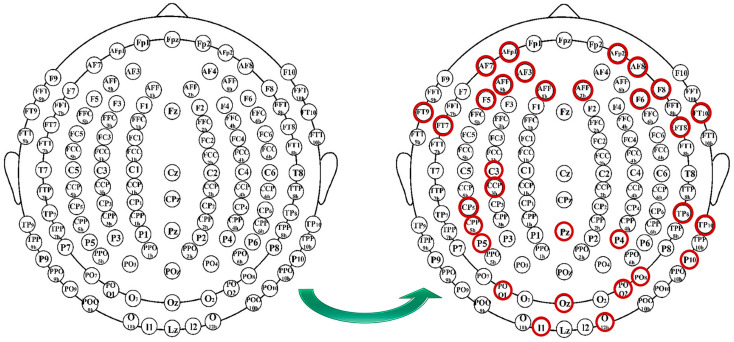
The most dominant features represented on EEG cap.

As shown in Figure 15 and Figure 16, these features were the most dominant feature on the output across all patients. Each feature represents a channel on ECI 128 channel Ag/AgCl electrode cap that affect the signal output. For example, the channel AFP1 was found 20 times in patients labeled “al” and “ay” and 18 times in patients labeled “aa.” The channel “F5” was found 20 times in patients labeled “aa” and “ay” and 16 times in patients labeled “av” and “aw.” The provided channels will assist the BCI community in utilizing them in their future work to consider these channels and use them in devices that will help the patients complete their tasks without assistance, such as a robotic arm or wheelchair.

The main advantages of the proposed work are:

- The proposed system exhibited a superior diagnostic capability, as evidenced by a high accuracy of 98.64%, a sensitivity of 98.8%, and a specificity of 98.5%.
- The system demonstrated robustness through its outstanding performance, evidenced by a high AUC of 98.4%.
- The proposed framework is entirely non-invasive in nature.
- The proposed framework provides an interpretation for the contribution of the selected feature sets to the probability of being class 1 or class 2.

The proposed work is not without its limitations. The following are identified as the primary drawbacks of this proposed work:

- Given the limited number of participants, with only five patients, it can be inferred that the sample size in the competition was small.
- All participants were confirmed to be free of neurological disorders and instructed to undertake the tasks assigned to them.
- The signal acquisition and preprocessing stages were omitted from the present work, as the data proprietors had already completed them.

The BCI clinical applications in real life:

- In reference [54], an attempt is made to assist patients in controlling hand opening-closing functions through orthosis, utilizing invasive and non-invasive techniques. However, it is acknowledged that further research is required in this area.
- As highlighted in reference [55], the proposed neurorehabilitation approach presents significant potential as a clinically successful therapy for restoring functional hand movement. However, it is stated that further validation is necessary through the conduct of carefully controlled studies on a larger patient population.
- As demonstrated in reference [56], this research indicates that a brain–computer interface (BCI) may serve as a practical substitute for a percutaneous bone-anchored hearing aid (BAHA). However, it is highlighted that additional studies are required to verify the effectiveness of this novel method both acoustically and medically.

We hope that the obtained results will help the BCI researchers develop a more robust and reliable system that will help the patients practice a normal life more effectively.

## 5. Conclusions

In conclusion, the proposed machine learning-based brain–computer interface (BCI) system exhibited exceptional accuracy, sensitivity, specificity, and area under the curve (AUC). The system’s overall accuracy was 98.64%, surpassing the majority of recent studies. Additionally, the system achieved an accuracy of over 99% for three out of the five patients. This achievement is attributable to the integration of the optimized whale optimization algorithm (WOA) feature selection technique, which effectively identified the optimal feature combinations with the greatest impact on the output and the modeled k-nearest neighbors (k-NN) machine learning classifier. In addition, LIME XAI technique was used to provide insights into the behavior of the suggested classification model by understanding the contribution of each selected feature towards the final classification. These promising results suggest that the proposed system has the potential to improve the quality of life and healthcare for patients and physicians by enabling the operation of a wide range of limb motor tasks. In future research, we intend to test further the proposed approach on additional datasets in real-world settings and explore its potential applications in a diverse range of limb motor tasks.

## Figures and Tables

**Table 1:** Summary of training and evaluation trails for the used dataset.

Subject	Training Trials	Evaluation of Trials
“aa”	168	112
“al”	224	56
“av”	84	196
“aw”	56	224
“ay”	28	252

**Table 2:** Obtained results by the proposed BCI approach on patient “aa”.

Classifier	Accuracy	Sensitivity	Specificity	AUC
k-NN	**0.98 ± 0.001**	**0.99 ± 0.001**	**0.98 ± 0.001**	**0.98 ± 0.001**
LDA	0.84 ± 0.005	0.84 ± 0.004	0.84 ± 0.01	0.84 ± 0.005
RF	0.97 ± 0.001	0.97 ± 0.001	0.97 ± 0.001	0.97 ± 0.001
LR	0.85 ± 0.007	0.84 ± 0.01	0.85 ± 0.01	0.85 ± 0.01
DT	0.85 ± 0.004	0.84 ± 0.004	0.85 ± 0.004	0.85 ± 0.004

**Table 3:** Obtained results by the proposed BCI approach on patient “al”.

Classifier	Accuracy	Sensitivity	Specificity	AUC
k-NN	**0.99 ± 0.0003**	**0.99 ± 0.0004**	**0.99 ± 0.0003**	**0.99 ± 0.0003**
LDA	0.94 ± 0.004	0.94 ± 0.005	0.94 ± 0.005	0.94 ± 0.005
RF	0.99 ± 0.0004	0.99 ± 0.001	0.99 ± 0.001	0.99 ± 0.0004
LR	0.94 ± 0.003	0.94 ± 0.003	0.94 ± 0.003	0.94 ± 0.003
DT	0.95 ± 0.003	0.95 ± 0.003	0.95 ± 0.002	0.95 ± 0.003

**Table 4:** Obtained results by the proposed BCI approach on patient “av”.

Classifier	Accuracy	Sensitivity	Specificity	AUC
k-NN	**0.99 ± 0.0004**	**0.99 ± 0.001**	**0.99 ± 0.0004**	**0.99 ± 0.0004**
LDA	0.94 ± 0.003	0.93 ± 0.003	0.94 ± 0.003	0.93 ± 0.003
RF	0.99 ± 0.001	0.98 ± 0.001	0.99 ± 0.001	0.99 ± 0.001
LR	0.94 ± 0.003	0.93 ± 0.003	0.94 ± 0.003	0.93 ± 0.003
DT	0.94 ± 0.003	0.93 ± 0.003	0.95 ± 0.002	0.94 ± 0.003

**Table 5:** Obtained results by the proposed BCI approach on patient “aw”.

Classifier	Accuracy	Sensitivity	Specificity	AUC
k-NN	**0.97 ± 0.001**	**0.98 ± 0.001**	**0.96 ± 0.001**	**0.97 ± 0.001**
LDA	0.83 ± 0.01	0.85 ± 0.013	0.81 ± 0.02	0.83 ± 0.014
RF	0.95 ± 0.001	0.98 ± 0.001	0.92 ± 0.002	0.95 ± 0.001
LR	0.83 ± 0.01	0.85 ± 0.01	0.81 ± 0.014	0.83 ± 0.013
DT	0.85 ± 0.004	0.85 ± 0.004	0.85 ± 0.004	0.85 ± 0.004

**Table 6:** Obtained results by the proposed BCI approach on patient “ay”.

Classifier	Accuracy	Sensitivity	Specificity	AUC
k-NN	**0.99 ± 0.0005**	**0.99 ± 0.001**	**0.99 ± 0.001**	**0.99 ± 0.001**
LDA	0.87 ± 0.004	0.87 ± 0.004	0.86 ± 0.004	0.87 ± 0.004
RF	0.98 ± 0.001	0.99 ± 0.001	0.97 ± 0.002	0.98±0.001
LR	0.86 ± 0.005	0.87 ± 0.005	0.86 ± 0.006	0.86 ± 0.005
DT	0.87 ± 0.004	0.88 ± 0.004	0.87 ± 0.003	0.88 0.004

**Table 7:** Classification performance comparison of the proposed BCI approach vs. other studies. The comparison was made on the same subjects, and the overall accuracy was calculated and compared, the best results of each study were bold.

Methods	Subjects					Accuracy	*p*-Value
	“aa”	“al”	“av”	“aw”	“ay”		
SGRM [51]	73.90	94.50	59.50	80.70	79.90	77.70	**0.0033**
Linear-SVM [12]	92.2	99.4	79.9	**98.9**	97.0	93.46	0.1513
NCFS [13]	90.00	98.93	76.71	98.21	97.14	92.20	0.1255
MSPCA, WPD, HOS and k-NN [16]	96	92.3	88.9	95.4	91.4	92.8	**0.0015**
TKCSP [17]	68.10	93.88	68.47	88.40	74.93	78.76	**0.0030**
AM-SVM [52]	86.61	**100.00**	66.84	90.63	80.95	85.00	**0.0245**
UDFS [53]	86.98	97.45	76.04	93.93	94.94	89.86	**0.0357**
**Proposed BCI approach**	**98.5**	99.48	**99.04**	97.15	**99.01**	**98.64**	---

## Data Availability

The data that has been used to conduct this study is accessible by the following link. https://www.bbci.de/competition/iii/desc_IVa.html (accessed on 9 December 2022).

## Supplementary Materials

The following supporting information can be downloaded at: https://www.mdpi.com/article/10.3390/s23063171/s1. The code for this research is available on GitHub at the following link: https://github.com/hend1011/bci-dataset-classification-.git (accessed on 12 March 2023). The code is released and can be used for research and educational purposes. We have attempted to make the code as user-friendly as possible, and have included detailed documentation and instructions for running the experiments. For further explanation of lime results please check Supplementary Files.

## Appendix A

**Table A1:** Comparison between the existing BCI systems on BCI Competition III dataset IVa.

Study	Feature Selection or Extraction	Classification Method	Results (%)	Findings
[12] (Attallah, Abougharbia et al., 2020)	Hybrid feature selection (CFS and CSE)	SVM LDA k-NN	93.46% 86.73% 88.7%	Techniques based on filters and wrappers are merged. The SVM classifier outperformed the other classifiers
[13] (Molla, Al Shiam et al., 2020)	UDFS NCFS	SVM LDA k-NN SVM LDA k-NN	89.86% 89.70% 89.14% 92.20% 91.36% 91.26%	SVM performs better than LDA and k-NN in every case. Despite the fact that LDA performs better with subject “aa” for NCFS. SVM has a much greater average accuracy than the other classifiers.
[14] (Joadder, Myszewski et al., 2019)	log variance RMS Decision tree median	LDA SVM mean k-NN	87% 78% 93% 99%	Using mean as a feature extraction method and K-NN as a classifier was the most effective combination for them to get the highest accuracy.
[15] (Baig, Aslam et al., 2017)	DE-based feature selection	LDA SVM	95.6% 95.6%	While all other subjects have classification accuracy above 90%, the subject “av” has the lowest classification accuracy of any by this method, at 88.9%.
[16] (Kevric and Subasi 2017)	MSPCA, WPD and HOS	k-NN	92.8%	For any of the five subjects, the suggested method does not provide the highest level of categorization precision. The single subject, “aa,” however, for which this suggested method obtains the highest categorization accuracy of 96%.
[17] (Dai, Zheng et al., 2018)	Transfer Kernel common spatial patterns	SVM	81.14%	a new approach to feature extraction called TKCSP that combines transfer kernel learning and kernel common spatial patterns (KCSP) (TKL) was used in this research
