# Supplementary figures and images for: An Integrated Machine Learning-Based Brain Computer Interface to Classify Diverse Limb Motor Tasks: Explainable Model
Source: Sensors (Basel). 2023 Mar 16;23(6):3171. doi: 10.3390/s23063171 (PMC10053613; doi:10.3390/s23063171)

### An example of 10 instances of class 1 on LIME:

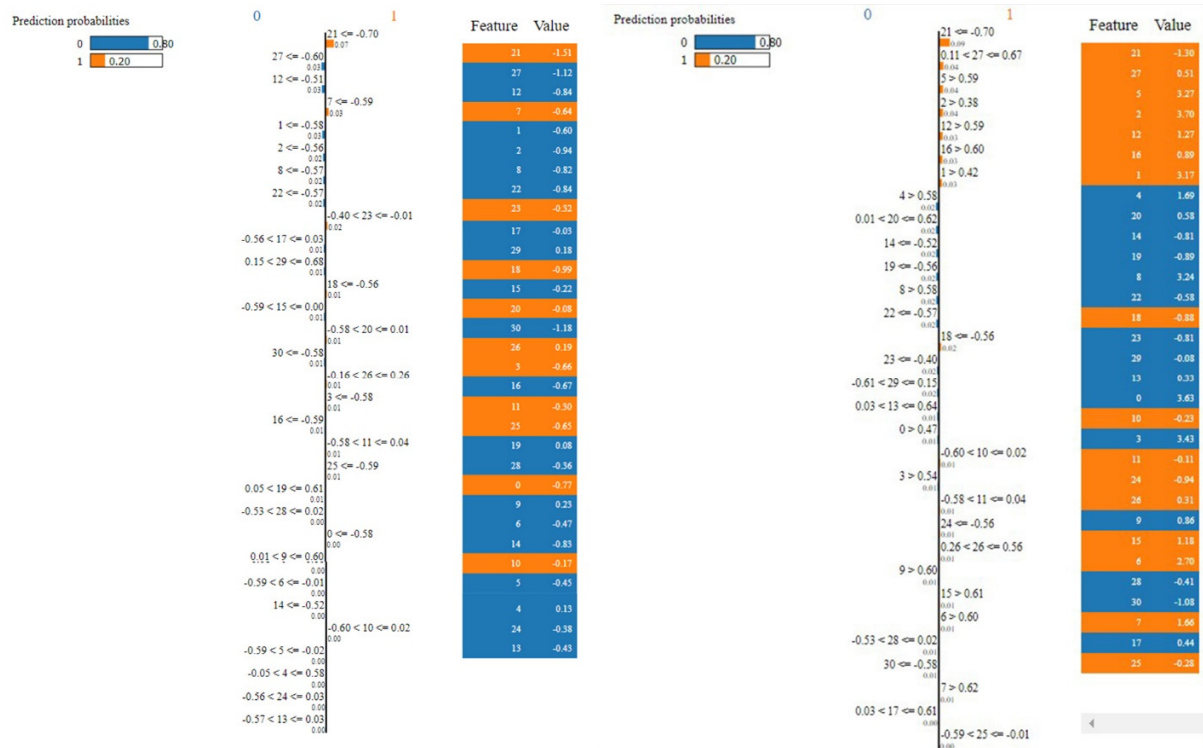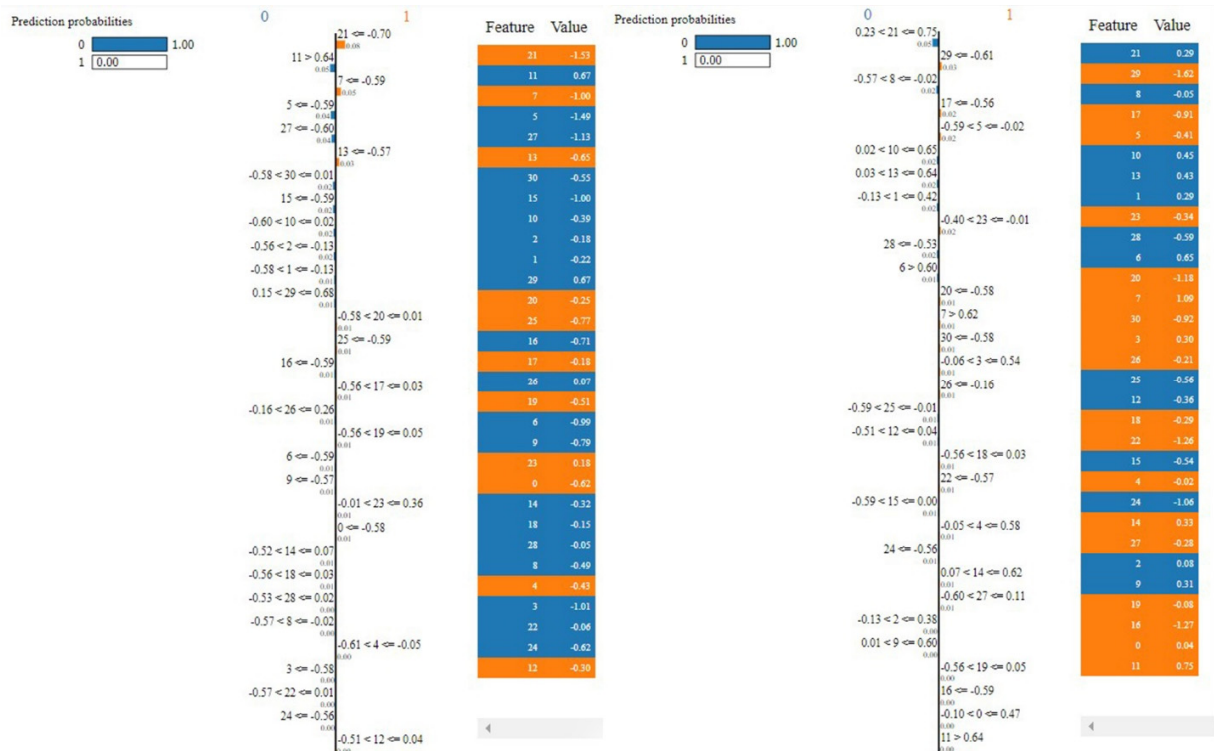

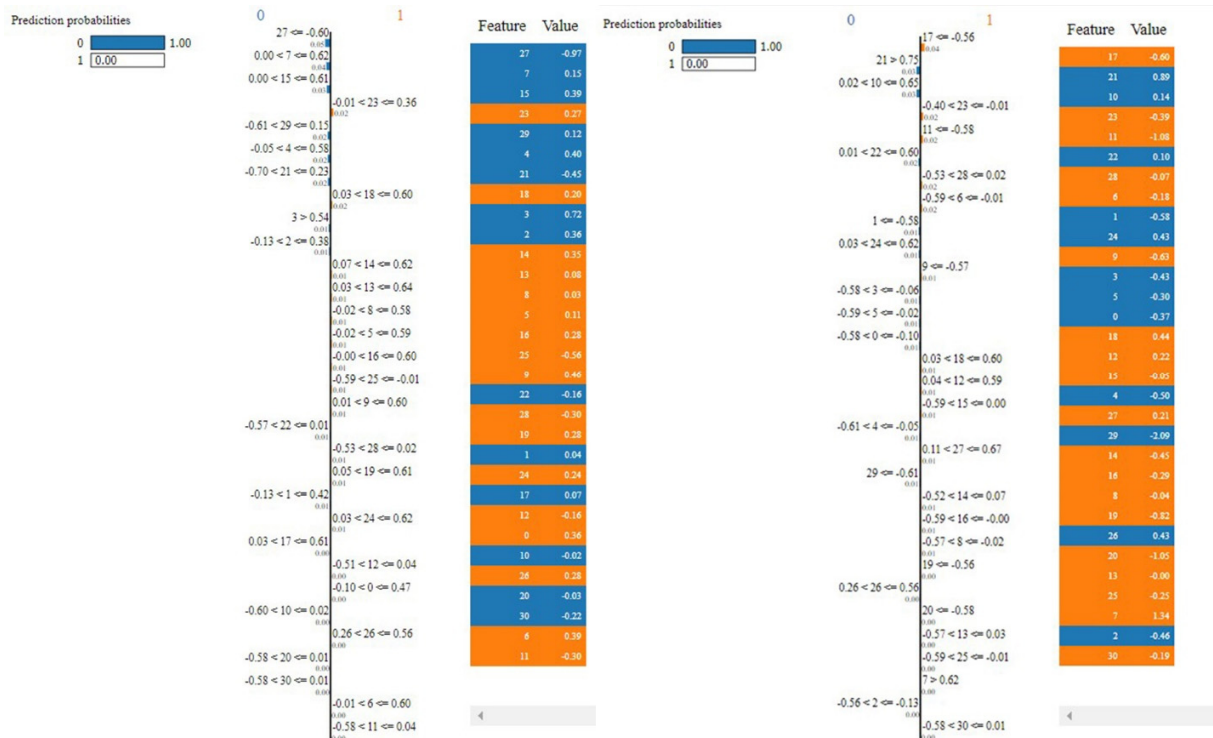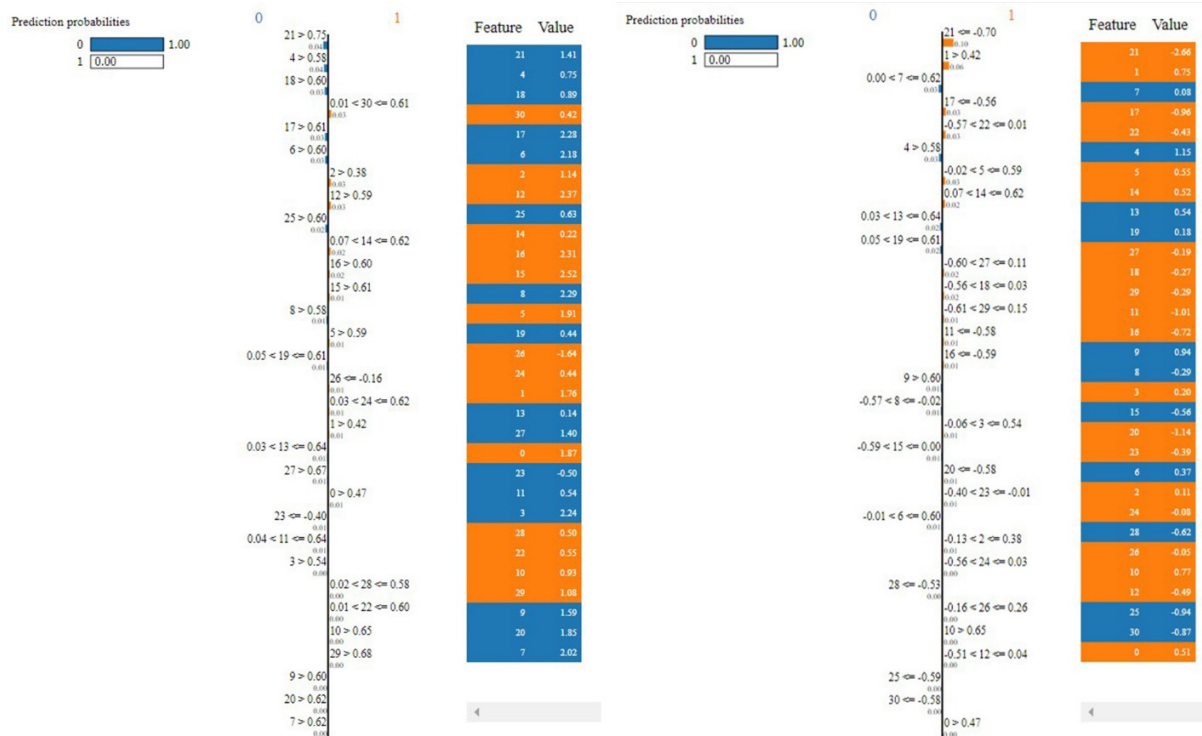

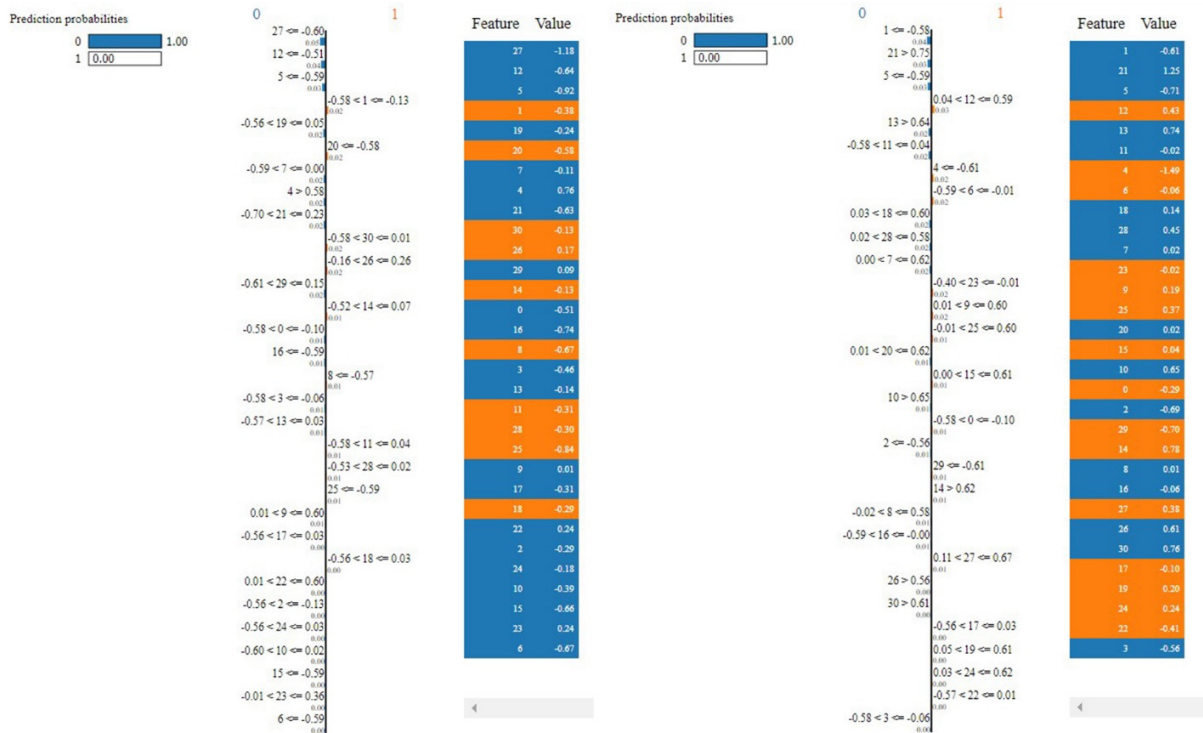

**An example of 10 instances of class 2 on LIME:**

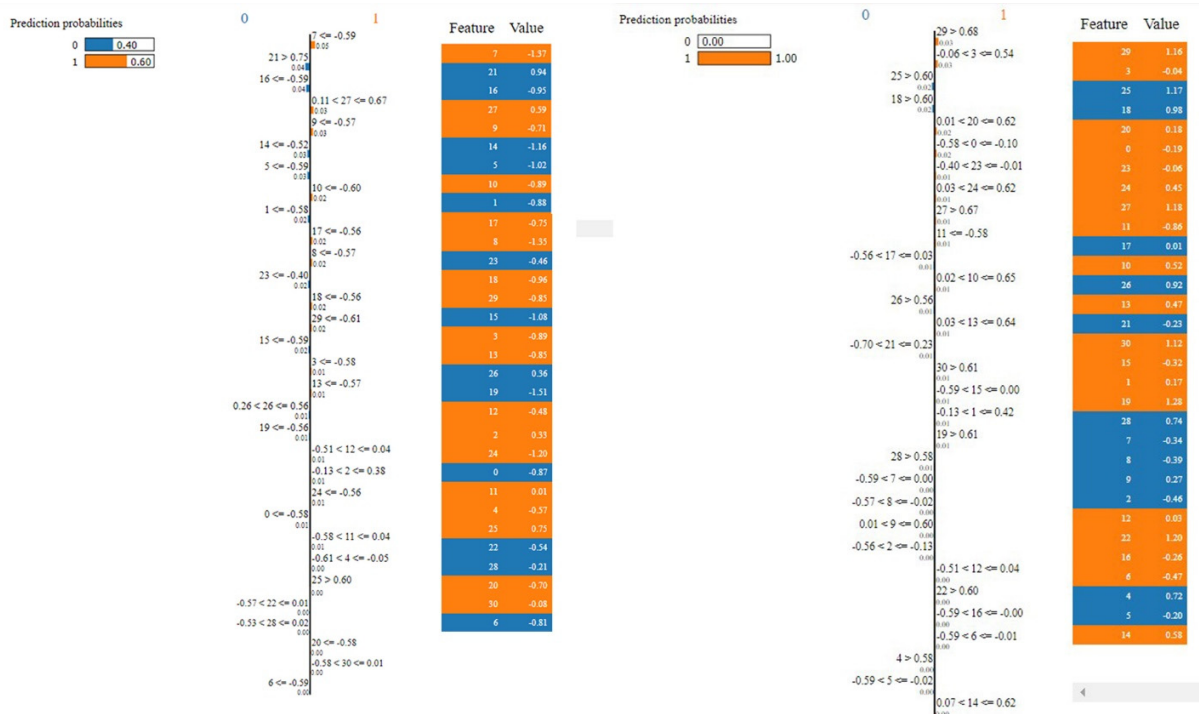

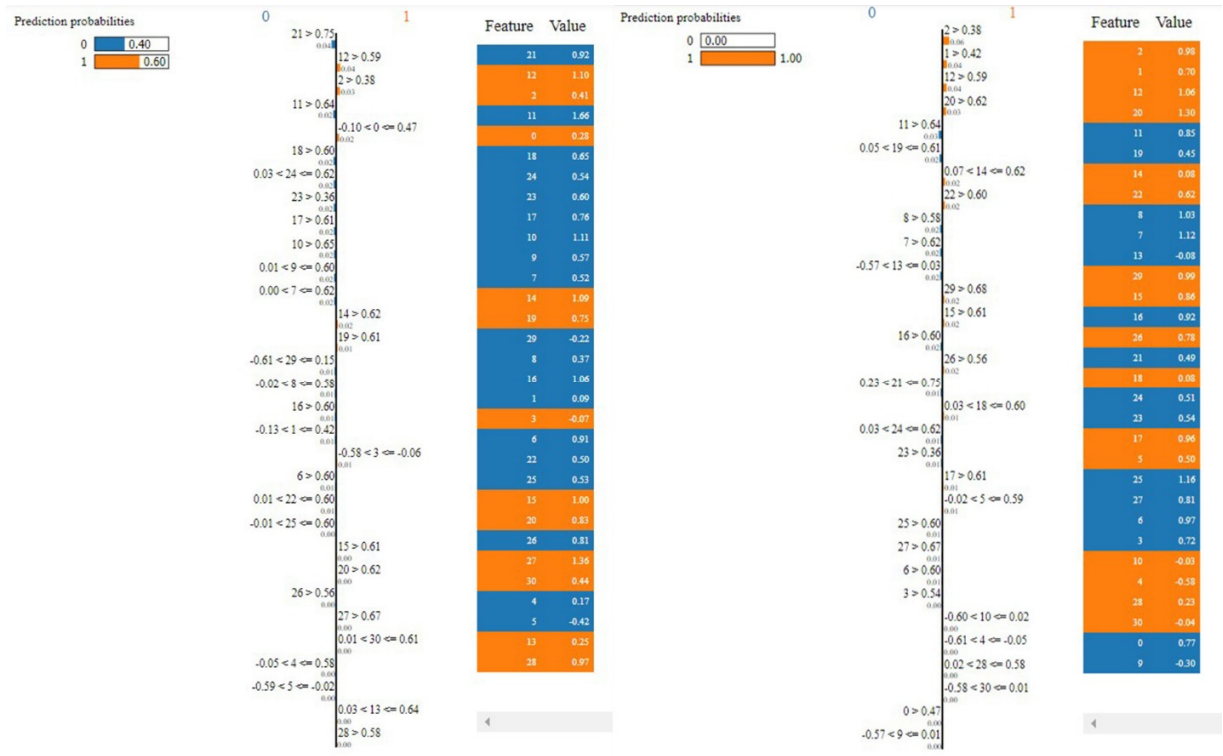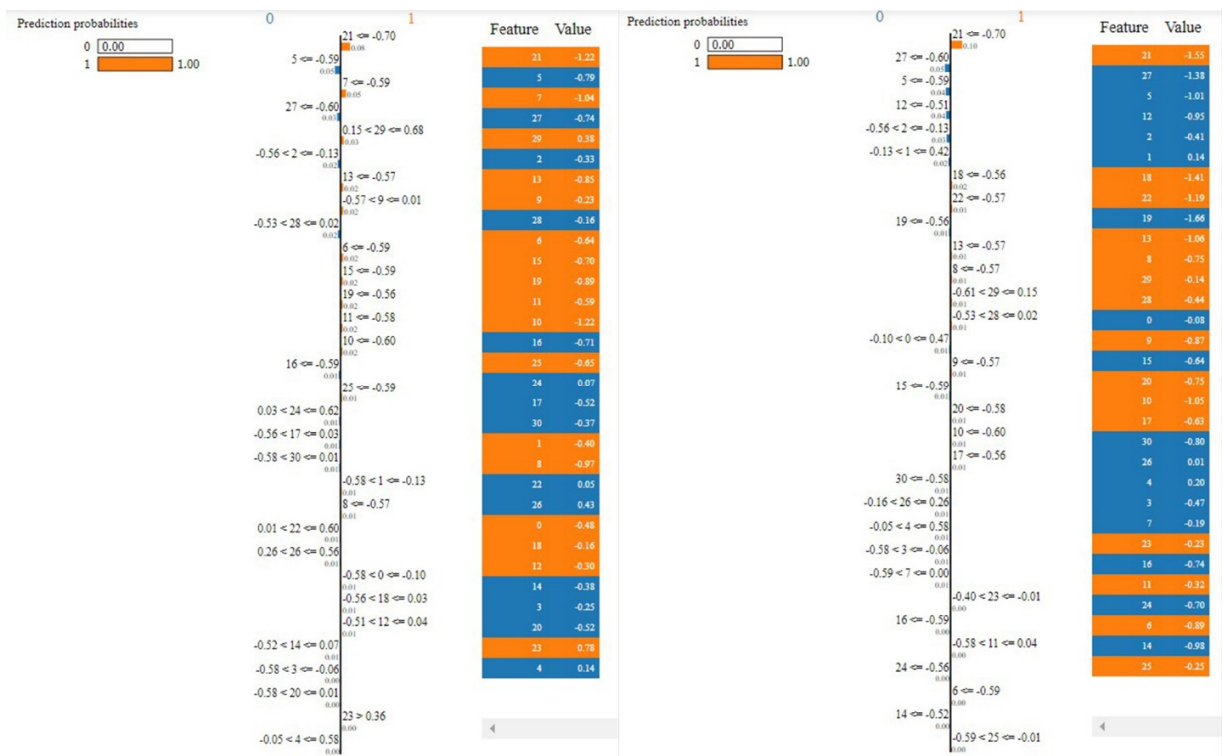

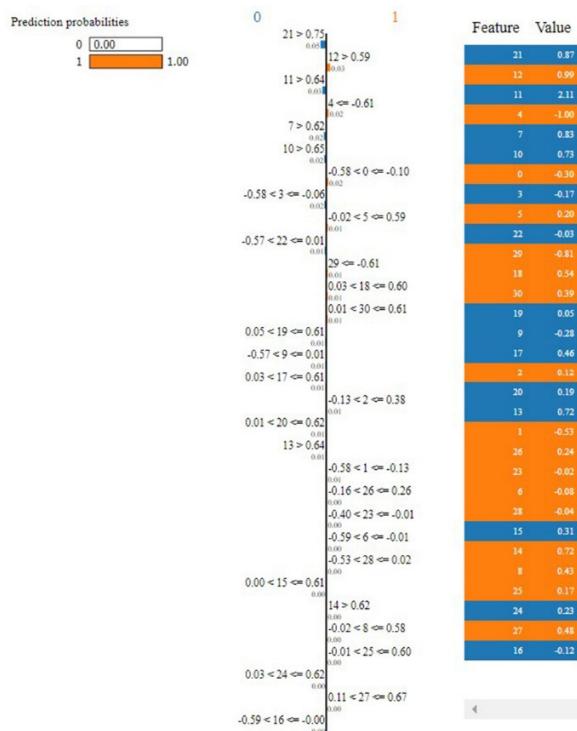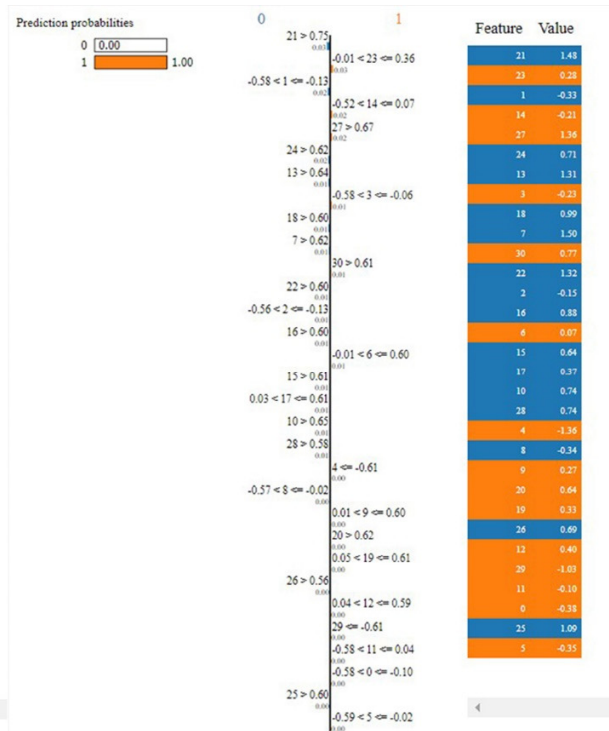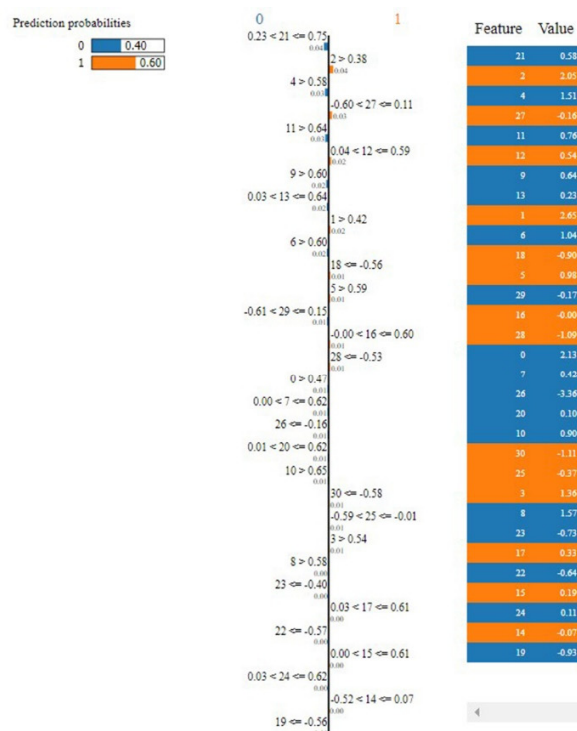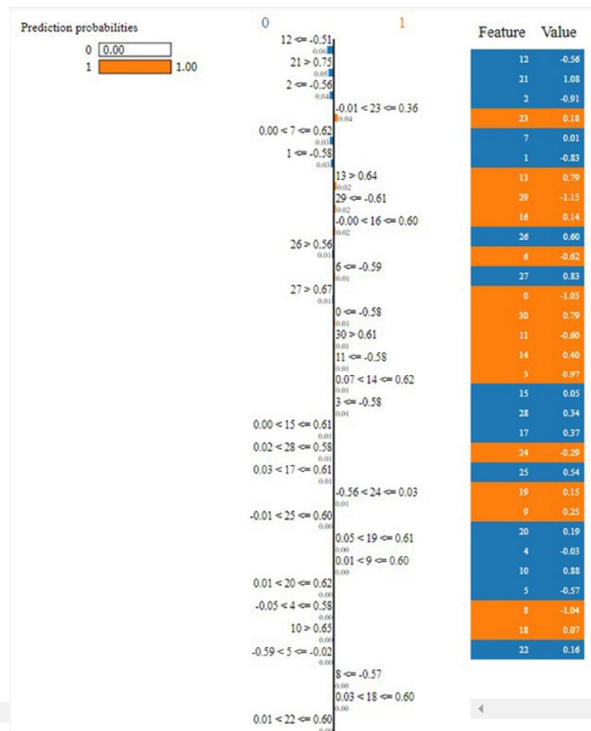

Supplement: Supplementary file 1 [file sensors-23-03171-s001.zip › SupFile S2 Detailed XAI Results.pdf]
